# Co-medication with disulfiram markedly increased serum clozapine levels: Two case reports

**DOI:** 10.1177/02698811221148611

**Published:** 2023-01-26

**Authors:** Lydia Hahl-Häkkinen, Susanna Maria Rask, Anssi Solismaa, Sanna Ruuhonen, Esa Leinonen

**Affiliations:** 1 Department of Psychiatry, Faculty of Medicine and Health Technology, Tampere University, Tampere, Finland; 2 Department of Psychiatry, Tampere University Hospital, Tampere, Finland

**Keywords:** Alcohol use disorder, clozapine, disulfiram, interaction, toxicity

## Abstract

**Background:**

Alcohol use disorder (AUD) is a significant co-morbidity in patients with schizophrenia. Clozapine offers some benefits in treating patients with refractory schizophrenia and AUD, but co-medicating with disulfiram is also common.

**Procedures:**

We report two cases where co-medicating with disulfiram led to a significant increase in clozapine serum levels.

**Findings:**

Clozapine serum levels decreased to one-third in Patient 1 when disulfiram was discontinued and started to increase again when disulfiram was reintroduced. Patient 2 developed toxic serum levels of clozapine during disulfiram treatment combined with heavy coffee drinking and symptoms reminiscent of neuroleptic malignant syndrome.

**Conclusions:**

Clozapine and disulfiram are both metabolized by cytochrome P450 CYP1A2 and clinically relevant interaction through this shared pathway is possible.

## Introduction

Alcohol use disorder (AUD) is a significant co-morbidity in patients with schizophrenia with worldwide prevalence of 24.3%, leading to increased hospitalization and morbidity (Lähteenvuo et al., 2021). Clozapine is the only approved medication for treatment-resistant schizophrenia with response rates around 30–50% in these patients (Kane et al., 2019). Moreover, clozapine has been reported to improve co-morbid AUD in schizophrenia patients, even leading to remission of substance abuse (Brunette et al., 2006). Pharmacotherapeutic interventions, including the use of disulfiram, have been recommended in patients with schizophrenia and co-morbid AUD (Akerman et al., 2014; Green et al., 2008). Co-medicating with disulfiram in clozapine patients is common in clinical settings, but reports on their potential pharmacological interactions are scanty (Brenner et al., 1994). Caffeine is a known inhibitor of clozapine metabolism through their shared cytochrome P450 CYP1A2 pathway, and drinking large amounts of coffee or other caffeinated drinks may cause clinically relevant increase in clozapine serum levels (Leon et al., 2022; Raaska et al., 2004; Yartsev and Peisah, 2021). Some medications increase clozapine serum levels through inhibition of CYP1A2 pathway, including fluvoxamine (Koponen et al., 1996) and ciprofloxacin (Brouwers et al., 2009).

We report two cases from the inpatient unit of the psychiatric department of Tampere University Hospital, Finland, with a considerable increase in clozapine serum levels when co-medicating with disulfiram. In Patient 2, caffeine also contributed to this interaction. To the best of our knowledge, clinically relevant interactions between clozapine and disulfiram have not so far been described elsewhere. Both patients gave written consent to the use of their records.

## Patients

Patient 1 is a 39-year-old woman of Caucasian origin. She had suffered from treatment-resistant schizophrenia since 2008 with epilepsy and AUD as comorbidities. She had been treated with clozapine 425 mg/d since May 2019 and the most recent serum clozapine level before initiation of disulfiram was 1200 nmol/l (392 ng/ml) and norclozapine 500 nmol/l (155 ng/ml). She had been taking valproate (established dose 2000 mg/d) for epilepsy for over 10 years. Her other medications were bisoprolol 2.5 mg/d, clobazam 20 mg/d, perphenazine decanoate 108 mg every 2 weeks long-acting injectable, multivitamin and magnesium medications. Prior to her transfer to our unit, disulfiram 200 mg/d treatment had been initiated to discourage alcohol use because her treatment was interrupted several times, due to her absconding from the hospital to consume alcohol. In November 2020, after transferring to our unit, the patient suffered a bout of bacterial pneumonia and was treated in our unit with per oram antibiotics. While keeping the clozapine daily dose constant during her pneumonia, serum clozapine level increased up to 2953 nmol/l (974 ng/ml) and norclozapine 800 nmol/l (248 ng/ml). One month after recovering from pneumonia, her clozapine serum level was still 2571 nmol/l (848 ng/ml) and norclozapine 817 nmol/l (253 ng/ml) with the same 425 mg/d dose. Her serum level of valproate was 541 µmol/l (7.8 mg/dl) on a 2000 mg/d dose. The dose of valproate had remained unchanged for years. Her clozapine and valproate serum levels were checked in April 2021, 4 months after this, without any changes in daily doses. Surprisingly, her serum clozapine level was markedly decreased and was only 788 nmol/l (260 ng/ml) and norclozapine 315 nmol/l (98 ng/ml). Her serum valproate level had remained constant at 517 µmol/l (7.5 mg/dl). The patient’s use of the medication was closely monitored. Nurses treating the patient and administering her medication did not suspect noncompliance with medication. The valproate levels remaining constant also supported this. Her mental state had not noticeably changed. All changes to patient’s medication were noted and potential changes in her daily habits were observed. The patient had not altered her smoking habits (15 cigarettes/d). Her caffeine intake was two cups of filtered coffee daily and had not changed. However, since the last clozapine concentration measurement 4 months ago, her disulfiram medication had been discontinued, bisoprolol medication had been changed to propranolol and allergy medication cetirizine had been started. It was suspected that changes in concomitant medication, especially disulfiram, might be the cause of the wide variation in her clozapine serum levels. After this speculation, the patient agreed to resume disulfiram treatment with a dose of 200 mg/d. Meanwhile, her clozapine dose was increased from 425 to 475 mg/day. Her clozapine serum level was monitored on day 2 of disulfiram rechallenge and was 1414 nmol/l (467 ng/ml) and norclozapine level was 508 nmol/l (157 ng/ml). On day 10, after the rechallenge of disulfiram, her clozapine serum level was 2127 nmol/l (702 ng/ml) and norclozapine level was 638 nmol/l (198 ng/ml) without any changes in clozapine dose. The patient started to suffer from night sweats and shivers and disulfiram was discontinued. Thereafter, her serum clozapine level decreased to 1182 nmol/l (390 ng/ml) and norclozapine level to 475 nmol/l (147 ng/ml) 10 days after discontinuation of disulfiram. The patient’s liver enzymes were normal throughout follow-up. The patient was tested for several pharmacogenetic genotypes (GeneAccount, Abomics; Pharmacogenetics Applied into Clinical Practice | Abomics, n.d.). Her CYP1A2 genotype was *1F/*1F meaning increased inducibility, CYP2D6 genotype was *1/*4 meaning partially slow metabolism, and normal metabolism in CYP2C19 and CYP3A4. The clozapine and norclozapine serum levels of Patient 1 are reported in Table 1.

**Table 1.** Clozapine and norclozapine serum levels of Patient 1 presented in chronological order from left to right with information on the clinical situation, dose of clozapine and whether disulfiram was in use.

	Before disulfiram initiation	After disulfiram initiation with pneumonia	Recovered from pneumonia	After disulfiram discontinuation	Two days after disulfiram rechallenge	Ten days after disulfiram rechallenge	Ten days after disulfiram discontinuation
Timeline in days	Day 0	Day 47	Day 77	Day 217	Day 258	Day 266	Day 280
Disulfiram in use	No	Yes	Yes	No	Yes	Yes	No
Clozapine dose, mg/d	425	425	425	425	475	475	475
Clozapine serum level, nmol/l (ng/ml)	1200 (392)	2953 (974)	2571 (848)	788 (260)	1414 (467)	2127 (702)	1182 (390)
Norclozapine serum level. nmol/l (ng/ml)	500 (155)	800 (248)	817 (253)	315 (98)	508 (157)	638 (198)	475 (147)

Patient 2 is a 26-year-old male of Caucasian origin admitted to our unit in August 2020, who had been diagnosed with paranoid schizophrenia one year prior and clozapine treatment had been initiated in July 2020. He also had a history of multiple substance abuse. He was a heavy smoker (20–40 cigarettes/d). In September, his clozapine dose was 400 mg/d and the corresponding serum level of clozapine was 728 nmol/l (240 ng/ml) and norclozapine 524 nmol/l (162 ng/ml). Disulfiram treatment was commenced 1 week later (in September) with 200 mg/d to discourage his urge to use alcohol and simultaneously his clozapine dose was increased to 650 mg/d. Two weeks after these changes in drug treatment, his clozapine serum level had risen to 2206 nmol/l (728 ng/ml) and norclozapine to 1048 nmol/l (325 ng/ml). Thus, the rise in serum clozapine level was threefold, although the clozapine dose was only increased 1.6-fold. His clozapine dose was decreased slightly to 600 mg/d and this yielded a clozapine serum level of 2133 nmol/l (704 ng/ml) and a norclozapine serum level of 1380 nmol/l (428 ng/ml). At that point, the patient’s regular co-medications were olanzapine 10–20 mg/d, chlorprotixen 25mg–100 mg/d, scopolamine 1 mg/72 h transdermal preparation (for excess salivation) and buprenorphine long-acting injectable 16 mg/1 week. His liver enzymes were monitored and were within the normal range. The patient began to experience worsening psychotic symptoms in mid-October; he was more agitated and had to be kept indoors. The staff noted that his coffee intake increased substantially up to several mugs of filtered coffee daily over a few days. Then, in late October, the patient started to vomit, complained of headache and sore throat, and was admitted to the emergency unit. He had received one injection of zuclopenthixol 50 mg intramuscularly one day before his admission to the emergency unit. His body temperature was slightly elevated to 37.3°C (99.1° Fahrenheit). His leukocyte count was 17.0 × 10^9^/l (reference range: 3.4–8.2 × 10^9^/l), C-reactive protein (CRP) was 11 mg/l (reference range: 0–10 mg/l), alanine transaminase was 18 U/l (reference range under 50 U/l) and gamma-glutamyl transferase was 29 U/l (reference range under 60 U/l), but his creatinine kinase level was 1416 U/l (reference range 50–400 U/l). Clozapine levels were checked when the patient was admitted to the emergency unit, but there was a delay of 6 days before these results were available. Neuroleptic malignant syndrome (NMS) was suspected and all other antipsychotics (except clozapine) were discontinued. Clozapine was continued with a reduced dose of 400 mg. The results of the serum clozapine levels received 6 days later were high; clozapine 5597 nmol/l (1830 ng/ml) and norclozapine 2750 nmol/l (899 ng/ml). Clozapine was discontinued immediately when these toxic levels were ascertained and disulfiram treatment was also discontinued in November. The patient’s liver enzymes began to rise after this but were normalized within a month. Clozapine was re-started slowly over several weeks, starting with a smaller dose and gradually increasing to a higher dose, which the patient tolerated without any complications. His caffeine intake, smoking and clozapine serum levels were constantly monitored and his latest dose of clozapine is 500 mg with serum levels of clozapine 1816 nmol/l (598 ng/ml) and norclozapine 797 nmol/l (247 ng/ml). Later, the patient received zuclopenthixol, chlorprotixen and olanzapine on several occasions without developing any signs of toxicity or NMS. Patient 2 was pharmacogenetically tested (GeneAccount, Abomics; Pharmacogenetics Applied into Clinical Practice | Abomics, n.d.) and he had CYP1A2 genotype *1F/*1F, meaning increased inducibility and normal metabolic findings for CYP3A4, CYP2C19 and CYP2D6. The clozapine and norclozapine serum levels of Patient 2 are reported in Table 2.

**Table 2.** Clozapine and norclozapine serum levels of Patient 2 presented in chronological order from left to right with information on the clinical situation, dose of clozapine and whether disulfiram was in use.

	Before disulfiram initiation	After disulfiram initiation	After clozapine dose decrease	Increased coffee intake, worsening of condition, suspected NMS	Later measurement (over 1 year later) in a stable condition
Timeline in days	Day 0	Day 21	Day 35	Day 51	Day > 365
Disulfiram in use	No	Yes	Yes	Yes	No
Clozapine dose mg/d	400	650	600	600	500
Clozapine serum level, nmol/l (ng/ml)	728 (240)	2206 (728)	2133 (704)	5597 (1830)	1816 (598)
Norclozapine serum level, nmol/l (ng/ml)	524 (162)	1048 (325)	1380 (428)	2750 (899)	797 (247)

## Discussion

We reported two cases where dramatic changes in clozapine serum levels occurred when co-medicating with two widely used, older drugs. As far as we know, no earlier reports of similar cases have been presented.

The Finnish Medicines Agency had no reports of similar incidences(Fimea–Etusivu, n.d.). According to the Eudravigilance database, there were no reports of a suspected interaction between clozapine and disulfiram. There were four reports of adverse effects of clozapine treatment in conjunction with altered clozapine serum levels (two elevated and two decreased) and concurrent use of disulfiram, but there was no information as to whether disulfiram had been initiated recently or had been in use for a longer period (European database of suspected adverse drug reaction reports, n.d.).

Clozapine metabolism is mediated by cytochrome P450 system, mainly by CYP1A2 (Raaska et al., 2004). The role of genetic polymorphisms in CYP1A2 and its effect on clozapine medication in clinical setting is poorly understood (Doude Van Troostwijk et al., 2003; Kootstra-Ros et al., 2005; Ruan and de Leon, 2020). The habitual clinical factors determining clozapine metabolism are age, ethnicity, sex, weight and smoking (De Leon et al., 2020; Haslemo et al., 2006) The first report that disulfiram may affect a metabolic pathway, which later was to be nominated as cytochrome P450 CYP1A2 system, was published in 1986, when it was reported that administering disulfiram to recovering alcoholics delayed their caffeine clearance by up to 30% when compared to healthy test subjects (Beach et al., 1986). Caffeine is metabolized by CYP1A2 system and is the only approved test substrate in studies on CYP1A2 metabolism (Perera et al., 2012). The effect of chronic disulfiram exposure on different cytochrome P450 systems has been studied later with the finding that disulfiram inhibits CYP2E1 in particular, but also has a wider effect on drug metabolizing enzymes (Frye and Branch, n.d.; Johansson, 1992; Poulsen et al., 1992). Interestingly, the potential of disulfiram to inhibit clozapine metabolism was speculated on in a letter to the editor in 1994, but the authors considered it more as a potential factor for increasing efficacy of treatment through increased clozapine serum levels (Brenner et al., 1994).

The effect of caffeine on clozapine metabolism is well known and several cases where caffeinated drinks have increased clozapine serum levels have been published (Raaska et al., 2004; Yartsev and Peisah, 2021). Other cases where the toxic effects of elevated clozapine serum levels have been confused with clinical picture of NMS have also been reported (Anand, 2012; Kamış et al., 2014). After discontinuing disulfiram, Patient 2 was rechallenged with other antipsychotics he had been treated with prior to his illness without any symptoms of NMS. This supports the conclusion that toxic serum levels of clozapine rather than the symptoms of NMS contribute to the illness leading to his admission to the emergency unit. NMS is likely to reoccur if patient is rechallenged with the same antipsychotic (Järventausta and Leinonen, 2000). As far as we know, there are no documented cases where NMS raised clozapine serum levels. Any inflammatory state that raises CRP may affect clozapine metabolism (Leon et al., 2022), but in Patient 2 CRP was low and he had no marked temperature elevation, thus making infection or inflammation an unlikely reason for such a significant elevation of clozapine serum levels.

In Patient 2, the caffeine consumption increased markedly over the few days before he developed clinical signs of clozapine toxicity. Disulfiram in addition to increased intake of caffeine likely contributed to raising clozapine levels in this patient. Disulfiram has also been shown to prolong the metabolism of caffeine (Beach et al., 1986). The prolonged metabolism of caffeine in addition to the increased intake could further increase the effect on clozapine levels. Both our patients were homozygotic carriers of genotype CYP1A2 *1F, the most common genotype in North European population (Nordmark et al., 2002; Söderberg et al., 2013). CYP1A2*1F is a single nucleotide polymorphism (rs762551) where C-allele is transitioned to A-allele, which is the more common variant worldwide in all populations according to the ALFA Allele Frequency Project (Phan et al., 2020). This genotype is connected with slower caffeine metabolism (Palatini et al., 2009).

However, although Patient 1 did not alter her coffee drinking habits, concomitant disulfiram medication more than doubled her clozapine serum levels. The highest clozapine concentrations of Patient 1 were measured after she developed pneumonia. Infections have been associated with elevated clozapine serum levels, possibly via the infection releasing cytokines that inhibit CYP1A2 (Clark et al., 2018). Serum levels remained high after Patient 1 recovered from pneumonia and she was later rechallenged with disulfiram, which led to clozapine serum levels again increasing, which supports our finding.

It can be speculated whether CYP1A2 *1F/*1F genotype, occurring in both our patients, is more sensitive to the inhibitory effects of disulfiram on CYP1A2 metabolism.

## Strengths and limitations

The strengths of our report are that we screened carefully for possible confounding factors and clozapine levels were measured several times over a period of time. We also screened the pharmacogenetic genotype of both patients.

A weakness of our report is that it consists only of two cases. We do not know the exact amount of coffee ingested by Patient 2, so the amount of caffeine causing the interaction can only be speculated.

## Conclusion

The cases reported together with earlier data on clozapine, disulfiram and caffeine metabolism suggest a newly found clinically relevant and potentially dangerous interaction between clozapine and disulfiram. Systematic investigations are needed to confirm this finding. Clinicians should be cautious if prescribing clozapine and disulfiram together.
